# Addressing urinary incontinence by gender: a nationwide population-based study in Turkiye

**DOI:** 10.1186/s12894-023-01388-2

**Published:** 2023-12-09

**Authors:** Melike Yavuz, Nilay Etiler

**Affiliations:** 1 Faculty of Medicine, Department of Public Health, Bahcesehir University, Istanbul, Türkiye; 2 School of Public Health, University of Nevada Reno, NV, USA; 3 Faculty of Medicine, Department of Public Health, Istanbul Okan University, Istanbul, Türkiye

**Keywords:** Urinary incontinence, Gender, Risk factors, Prevalence, Population-based study

## Abstract

**Background:**

Urinary incontinence (UI), which usually occurs in women but affects both sexes, is a significant public health challenge. This study aims to comprehensively investigate the prevalence and determinants of UI in men and women, considering gender-specific factors.

**Methods:**

The study performed a secondary analysis on data obtained from 13,383 individuals surveyed in the 2019 Turkish Health Survey, providing a representation of the Turkish population. The dataset included sociodemographic and health-related variables like UI, body mass index (BMI), physical activity, smoking, and chronic diseases—statistical analysis employed chi-square tests and gender-stratified logistic regression models to identify UI-associated factors.

**Results:**

Our results showed that UI affected 8.8% of the population, with a striking gender disparity. Women had a notably higher prevalence at 11.2%, while men had a lower rate of 5.5%. Importantly, this gender gap narrowed with age. For example, in the 34–44 age group, the female/male ratio was 6.9, but it decreased to 1.4 in the 65–74 age group. Marital status and employment status played significant roles. Separated, divorced, or widowed individuals, particularly women, had the highest prevalence at 19.3%. Employment status influenced UI prevalence, with employed men having the lowest rate (2.1%), while retired women faced the highest rate (15.0%). Higher BMI, especially in obese individuals, significantly raised UI prevalence, reaching 7.9% for men and 15.8% for women. Physical inactivity, notably in women (17.0%), and prolonged sedentary hours (13.9%) were associated with higher UI rates. Former smokers, especially women (15.9%), had a notable impact on UI. Poor perceived health and chronic conditions like Chronic Obstructive Pulmonary Disease (COPD), hypertension, and diabetes were significantly associated with higher UI prevalence. Logistic regression analysis revealed that age, education, perceived health status, COPD, and diabetes were significant factors associated with UI in both sexes, while in women, BMI, physical activity, and smoking also played notable roles.

**Conclusions:**

This extensive UI study has unveiled notable gender disparities and determinants. Notably, these disparities decrease with age, underlining UI’s changing nature over time. Modifiable factors impact women more, while non-modifiable factors are linked to men. The study underscores the importance of tailoring healthcare strategies to address UI based on gender.

## Background

Urinary incontinence (UI), defined as involuntary leakage of urine, is a common and often underestimated health problem with a significant impact on quality of life [1]. Although traditionally associated with women, research suggests that UI affects both sexes, with different prevalence and determinants in different populations [2]. As in many other countries, UI is a significant public health problem in Turkiye since it can affect daily activities, social interactions, and psychological well-being [3, 4]. Understanding the prevalence and determinants of UI is critical for developing effective health strategies and interventions tailored to the unique characteristics of the population.

Urinary incontinence can manifest as stress, urge, or mixed, with each form having different causes and manifestations [1]. Stress incontinence, the most common form, is the complaint of involuntary leakage on effort or exertion or on sneezing or coughing. Urge incontinence, on the other hand, is accompanied by or immediately preceded by urgency.

Although extensive research has been conducted on UI, many studies focus predominantly on women and often neglect the potential impact on men. Moreover, the gender determinants of UI in certain cultural contexts, such as Turkiye, have not been adequately explored. To address these gaps, this study aims to comprehensively examine the prevalence and determinants of UI in Turkiye, considering gender differences.

This study aims to shed light on the prevalence and determinants of UI in Turkish men and women and identify sociodemographic, health-related, and lifestyle factors that contribute to its occurrence. By understanding the multifaceted nature of UI, healthcare professionals and policymakers can develop targeted interventions that address the various factors that influence the prevalence of the disease.

## Methods

### Study design and data sources

To investigate the frequency and potential factors associated with urinary incontinence in Turkiye, we conducted a secondary analysis of microdata from the 2019 Turkish Health Survey (THS), a cross-sectional study conducted by the Turkish Statistical Institute (TurkStat). We requested the 2019 Health Survey micro data set from TurkStat within the official procedure on use of microdata sets of the institute (For further information: https://www.tuik.gov.tr/Kurumsal/Mikro_Veri/En).

### Population and sampling

The survey is intended to provide estimates for the total population of Turkey. Accordingly, the total sample size was set at 9,470 household addresses. A two-stage stratified cluster sampling procedure is used, with the urban/rural distinction serving as the criterion for external stratification. The first-stage sampling unit consists of blocks randomly selected in proportional size from clusters with an average of 100 household addresses. The second-stage sampling unit consists of systematically randomly selected household addresses from each selected cluster. The survey sample doesn’t cover the individual residing in correctional facilities, military quarters, or nursing homes. Additionally, participants aged 75 and older were excluded to enhance the accuracy and reliability of the reported data. Furthermore, individuals lacking weight and height information were also excluded from the study.

This sample included 13,383 individuals (6038 men and 7345 women) aged 15 to 74 years, representing a broad spectrum of the Turkish population.

### Data collection method

Within the scope of the health survey, data was collected from individuals residing in sampled households through the computer-assisted personal interviews (CAPI) method, which were conducted face-to-face. This method allowed direct contact with participants and contributed to the reliability of the information obtained. The interviews were carried out between September and December of 2019.

### Definitions and classification of data

The variable of primary interest in this study was IU, which was measured by the question, “In the past 12 months, have you had urinary incontinence or problems controlling your bladder?” Independent variables included sociodemographic factors such as age, education, marital status, employment, and household type. In addition, health-related factors such as body mass index (BMI), physical activity, smoking status, perceived health status, and the presence of chronic diseases such as diabetes, chronic obstructive pulmonary diseases (COPD), and hypertension were considered. BMI calculations were based on self-reported weight (kg) and height (cm).

Responses on perceived health status were categorized as “excellent or good,” “fair,” and “poor or very poor.” The presence of chronic conditions (asthma, COPD, diabetes) was included in the analysis by participants’ self-report of having suffered from these conditions in the past 12 months.

Physical activity levels were assessed by the frequency of continuous 10-minute walks during a standard week, with responses categorized as none, 1–3 days, or 4–7 days per week. Sedentary behavior was assessed by daily sitting and resting time, categorized as less than 4 h (active), 4–6 h, or more than 6 h (sedentary). Smoking status and BMI were respectively categorized as current smoker, former smoker, nonsmoker, and less than 25 kg/m^2^(underweight and normal), 25–29.9 kg/m^2^ (overweight), ≥ 30 kg/m^2^ (obese).

### Ethical considerations

The microdata set utilized in this study was procured following the official procedure outlined by TurkStat, ensuring compliance with established protocols and ethical considerations. Since we conducted a secondary analysis of an existing microdata set from an established survey, ethical approval was not required for our research.

### Statistical analysis

After the microdata set was obtained, missing data on the study variables were excluded from the microdata set. Bivariate analyses were conducted using chi-square tests, and multivariate analysis was performed using a backward stepwise logistic regression model. Notably, these analyses were carried out separately for men and women, recognizing potential gender-based differences. For all analytical procedures, 25.0 (SPSS Inc., Chicago, IL, USA) was utilized as the software platform.

## Results

**Fig. 1:**
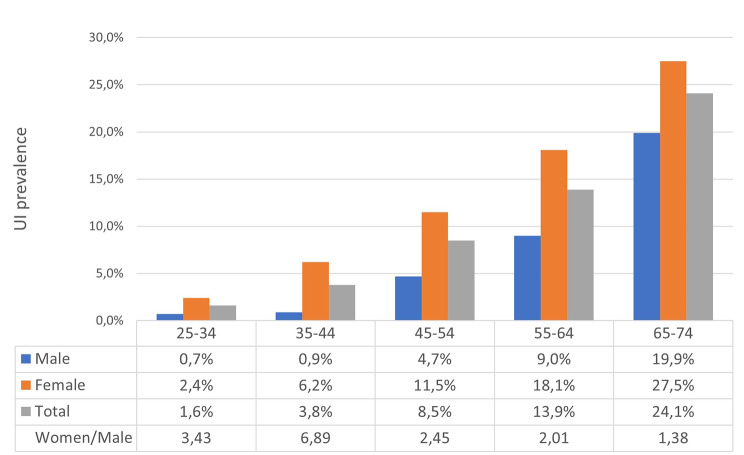
Prevalence of urinary incontinence according to age and gender groups

Among a total of 13,383 participants, the overall prevalence of urinary incontinence (UI) was 8.8% (n = 1172). This prevalence varies significantly between genders: Women have a significantly higher prevalence of 11.2% (n = 835), while men have a comparatively lower rate of 5.5% (n = 337) (*p* < 0.001). Figure 1 shows a direct correlation between the prevalence of UI and advancing age. It is noticeable that the difference between genders decreases with age. The largest difference is observed in the age group 34–44 years, while the age group 65–74 years shows the smallest differences.

Table 1 provides a comprehensive overview of urinary incontinence prevalence based on various categories, such as marital status, education, employment, and household type, for both men and women. It is evident that the prevalence of UI varies considerably between different marital statuses. Singles have the lowest UI prevalence, whereas individuals who are separated, divorced, or widowed have the highest prevalence, with women showing a particularly marked disparity in this category. The statistically significant differences underscore the role of marital status in predicting the prevalence of UI. Employed individuals, especially men, have the lowest UI prevalence (n = 98; 2.5%), whereas retired individuals, especially women, have the highest prevalence (n = 295; 15.0%). The prevalence is significantly higher in the non-employed group of both sexes and the whole group, including housemakers. The significant differences highlight the possible influence of occupation and lifestyle on the prevalence of UI. Different household compositions yield varying UI prevalence rates. For example, single-person households and nuclear families tend to have lower UI prevalence. In contrast, households with only one spouse or single parents and children tend to have higher UI prevalence. The significant differences suggest that household structure may be a relevant factor influencing UI prevalence.

**Table 1:** The relationship of UI with social characteristics among men and women

	Men		Women		Overall	
	**No.** ***†***	**UI (%)**	**No.** ***†***	**UI (%)**	**No.** ***†***	**UI (%)**
**Marital status**						
Single	730	1.5%	500	2.8%	1230	2.0%
Married	5071	6.0%	5815	10.4%	10,886	8.4%
Separated/divorced/widowed	237	8.0%	1030	19.3%	1267	17.2%
Sig.		*< 0.001*		*< 0.001*		*< 0.001*
**Education**						
≤ 8 yrs	3436	8.1%	5047	14.3%	8483	11.8%
9–12 yrs	1232	2.1%	1040	5.8%	2272	3.8%
> 12 years (college, faculty bachelor, and higher)	1370	1.9%	1258	2.9%	2628	2.4%
Sig.		*< 0.001*		*< 0.001*		*< 0.001*
**Employment**						
Employed	3947	2.5%	1777	6.0%	5724	3.6%
Not employed (including housemakers)	627	5.9%	5063	12.1%	5690	11.4%
Retired	1464	13.3%	505	20.0%	1969	15.0%
Sig.		*< 0.001*		*< 0.001*		*< 0.001*
**Household type**						
One person household	322	4.3%	475	19.6%	797	13.4%
Only spouses (2 persons)	1207	10.3%	1282	15.2%	2489	12.8%
The nuclear family (Couple and children)	3269	3.8%	3636	8.0%	6905	6.0%
At least one nuclear family and others	814	5.9%	1063	12.1%	1877	9.4%
More than one person without a nuclear family	184	4.9%	273	11.0%	457	8.5%
Single parents and children	242	5.8%	616	13.5%	858	11.3%
Sig.		*< 0.001*		*< 0.001*		*< 0.001*
**Total**	**6038**		**7345**		**13,383**	

*† Total counts for each group*

Table 2 shows the relationship between various health and lifestyle factors and the prevalence of UI. It is noteworthy that the prevalence of UI increases with higher BMI categories. Obese individuals (BMI ≥ 30) have the highest UI prevalence, both in men and women (n = 102; 7.9% and n = 340; 15.8%, respectively). The differences are statistically significant and highlight the association between higher BMI and a higher likelihood of developing UI. When examining the association between regular physical activity, specifically walking, and the prevalence of UI, it is clear that more physical activity is associated with a lower prevalence of UI. Specifically, women who reported being physically inactive had the highest prevalence of UI (n = 244; 17.0%). In the context of sedentary behavior, longer sedentary hours correlated with higher UI prevalence. Women who spend more time in sedentary activities demonstrate greater UI prevalence (n = 358; 13.9%). The significant differences underscore the importance of staying active to mitigate the risk of UI.

**Table 2:** The relationship of UI with health-related characteristics among men and women

	Men		Women		Overall	
	**No.** ***†***	**UI (%)**	**No.** ***†***	**UI (%)**	**No.** ***†***	**UI (%)**
**Body mass index**						
Underweight and normal (< 25.0)	2000	5.3%	2672	5.6%	4672	5.4%
Overweight (25.0-29.9)	2740	4.6%	2520	10.3%	5260	7.3%
Obese (≥ 30)	1298	7.9%	2153	15.8%	3451	14.9%
Sig.		< 0.001		< 0.001		< 0.001
**Physical Activity / Walking**						
None	763	8.9%	1436	17.0%	2199	14.2%
1–3 days	796	6.7%	1639	13.4%	2435	11.2%
4–7 days	4479	4.7%	4270	8.3%	8749	6.5%
Sig.		0.056		< 0.001		< 0.001
**Sedentary living**						
< 4 h (active)	2220	3.6%	2790	8.8%	5010	6.5%
4 to 6 h	1892	4.3%	1975	10.9%	3867	7.7%
6 h and highest (sedentary)	1926	8.9%	2580	13.9%	4506	11.7%
Sig.		< 0.001		< 0.001		< 0.001
**Smoking**						
Currently smoker	2862	3.7%	1601	10.9%	4463	6.3%
Non-smoker	1584	4.7%	5078	10.6%	6662	9.2%
Former smoker	1592	9.4%	666	15.9%	2258	11.3%
Sig.		< 0.001		< 0.001		< 0.001
**Perceived health (self-rated health)**						
Excellent or Good	3840	1.6%	3614	3.7%	7454	2.6%
Fair	1727	9.1%	2759	15.1%	4486	12.8%
Poor or very poor	471	24.0%	972	27.7%	1443	26.5%
Sig.		< 0.001		< 0.001		< 0.001
**COPD, chronic lung diseases**						
Yes	360	18.3%	747	23.7%	1107	22.0%
No	5678	4.7%	6598	9.7%	12,276	7.4%
Sig.		< 0.001		< 0.001		< 0.001
**Hypertension**						
Yes	867	15.5%	1734	22.4%	2601	20.1%
No	5171	3.8%	5611	7.7%	10,782	5.8%
Sig.		< 0.001		< 0.001		< 0.001
**Diabetes**						
Yes	641	16.7%	1083	24.5%	1724	21.6%
No	5397	4.2%	6262	8.9%	11,659	6.7%
Sig.		< 0.001		< 0.001		< 0.001
**Total**	**6038**		**7345**		**13,383**	

*† Total counts for each group*

The relationship between smoking habit and the prevalence of UI is also presented in Table 2. Former smokers, particularly women (n = 105; 15.9%), tend to have higher UI prevalence. The prevalence is lower among non-smokers, and current smokers exhibit intermediate levels. The statistical significance indicates that smoking may contribute to increased UI prevalence.

Table 2 also examines the effects of self-perceived health on the prevalence of UI. The results show that individuals who rate their health as fair or poor/very poor are more likely to experience the UI. The statistically significant differences underscore the association between perceived health status and UI, suggesting that addressing overall health and well-being might influence the occurrence of UI.

The data presented in Table 2 show a remarkable association between chronic health conditions (COPD, hypertension, and diabetes) and the prevalence of UI. Participants with COPD have a substantially higher prevalence (n = 243; 22.0%) of UI compared to those without COPD (908; 7.4%). This holds true for both men and women. The statistically significant differences in UI prevalence between COPD and non-COPD groups underscore the potential impact of respiratory health on UI occurrence. The prevalence of UI exhibited a notable increase among individuals diagnosed with hypertension (n = 522; 20.1%) compared to those without this condition (n = 625; 5.8%). This pattern remains consistent among both men and women. These observed differences in prevalence carry statistical significance, highlighting the association between hypertension and an elevated probability of encountering UI. Similar to the previous two conditions, participants with diabetes also present a heightened prevalence of UI. Notably, the prevalence stands at 21.6% (n = 372) among those with diabetes, in contrast to 6.7% (n = 781) among those without the condition (*p* < 0.001). This is true for both men and women.

Table 3 presents results from a logistic regression analysis examining the association between various factors and UI. The Nagelkerke R^2^ values indicate the model’s goodness of fit. These results indicate that in women, UI is notably associated with age, education, BMI, perceived health status, COPD, diabetes, physical activity, and smoking. In men, UI is linked significantly to age, education, perceived health status, COPD, and diabetes.

**Table 3:** Multi-variable analysis of factors affecting UI by men and women

	*Male*			*Female*		
**Nagelkerke R** ^**2**^		0.286			0.216	
	**Sig.**	**OR**	**CI 95%**	**Sig.**	**OR**	**CI 95%**
**Age groups**						
Ref: 25–34		1.00			1.00	
35–44	NS	NS	-	0.003	1.77	1.21–2.58
45–54	< 0.001	3.24	1.62–6.49	< 0.001	2.39	1.66–3.46
55–64	< 0.001	5.40	2.74–10.66	< 0.001	3.51	2.43–5.07
65–75	< 0.001	10.58	5.36–20.89	< 0.001	5.37	3.68–7.84
**Education**						
Ref: Collage, faculty, and higher		1.00			1.00	
≤ 8 yrs	0.013	1.74	1.13–2.68	< 0.001	1.98	1.37–2.85
9–12 yrs	NS	NS	-	NS	NS	-
**BMI Groups**						
Ref: <25.0		1.00			1.00	
Overweight (25.0-29.99)	NS	NS	-	0.027	1.29	1.03–1.61
Obese ( > = 30.0)	NS	NS	-	< 0.001	2.00	1.61–2.49
**Perceived health status**						
Ref: Excellent/Good		1.00			1.00	
Fair	< 0.001	3.01	2.19–4.15	< 0.001	2.54	2.05–3.14
Poor / Very poor	< 0.001	6.89	1.78–9.92	< 0.001	3.95	3.07–5.07
**COPD**	0.001	1.72	1.23–2.39	0.008	1.32	1.08–1.62
**HT**	NS	NS	-	NS	NS	-
**Diabetes**	0.007	1.47	1.11–1.95	0.002	1.33	1.11–1.60
**Sedentary living**	NS	NS	-	0.028	1.22	1.02–1.46
**Walking**						
Ref: 4–7 days a week		1.00			1.00	
None	NS	NS	-	0.003	1.35	1.11–1.64
1–3 days a week	NS	NS	-	0.012	1.28	1.06–1.62
**Smoking**						
Ref: non-smoker		1.00			1.00	
Currently smoker	NS	NS	-	< 0.001	1.65	1.35–2.03
Former smoker	NS	NS	-	< 0.001	1.68	1.31–2.15
Constant	< 0.001		-5.426	< 0.001		-5.327

*NS: Not significant*

## Discussion

In this secondary analysis of a cross-sectional study conducted on a large representative sample from Turkiye, our investigation focused on identifying the determinants of UI in men and women separately. UI is often considered a problem that primarily affects women, which leads most studies to focus on female populations. Our study found that the overall prevalence of UI in Turkiye is approximately 9%, with a significantly higher prevalence in women (11%) than in men (5.5%). A consistent theme runs through our results, highlighting the significant influence of gender on the prevalence of UI. This underscores the importance of gender-sensitive healthcare strategies and interventions when addressing UI.

Our study discloses that UI is also a pertinent health issue for men, with its prevalence approaching a similar frequency in both genders as age advances. Notably, the widest gap in UI prevalence was observed within the age group of 34–44 years, while the smallest gap was apparent in the age bracket of 65–74 years. Prevalence started increasing after 45 years among men and after 35 years among women. This finding harmonizes with prior studies, including Unlu et al.‘s [5] research, which associated an age over 35 with an 89.6% rise in UI prevalence. Age consistently emerges as a contributing factor in the likelihood of UI across numerous studies [4, 6, 7]. In a recent study of hospitalized patients in Turkiye, the prevalence of UI in 1176 hospitalized patients was 29.4%, whereas this rate increased to 41.6% in patients over 65 years of age [7].

Another noteworthy determinant unveiled by our study was educational level. A lower educational attainment increased the risk for UI in both sexes. Interestingly, the risk associated with lower educational levels was more pronounced in women, with a 1.98-fold increase, compared to a 1.74-fold increase in men. The impact of education on UI is complex, as evidenced by Grzybowska et al.‘s finding that higher education levels correlated with a decreased likelihood of using incontinence pads constantly [8]. Similarly, a study on Estonian postmenopausal women indicated that secondary education correlated with an 87% increase in UI odds [9].

The present study’s employment status findings highlight significant differences in UI prevalence among men and women in various employment categories. Men demonstrated lower UI prevalence when employed, whereas women exhibited a higher prevalence. Conversely, UI prevalence increased among both genders when unemployed or retired. These results emphasize the importance of considering employment status as a contributing factor in UI prevalence and highlight the need for tailored interventions to address UI within different employment categories.

Smoking’s influence on UI is multi-faceted and persistent coughing, often associated with smoking, can exert pressure on pelvic muscles, potentially weakening them and increasing the risk of stress incontinence [10]. Furthermore, smoking’s bladder-irritating effects can lead to more frequent bathroom visits [11]. Additionally, smoking has been linked to bladder cancer [12, 13]. In our study, male smoking didn’t show a significant association with UI, yet female current and former smokers exhibited higher UI prevalence than non-smokers. These findings align with research suggesting that current and former smoking heightens stress and motor incontinence in women [14]. However, our results diverge from studies that established a strong smoking-related association with lower urinary tract symptoms in men [15, 16].

In our study, a relationship was found between self-perceived health and the prevalence of UI. Those who reported fair or poor/very poor health were significantly more likely to suffer from UI. These findings highlight the association between perceived health status and UI, implying that improving overall well-being could be related to UI. This highlights the potential of comprehensive health strategies to mitigate UI risk by addressing individual health perceptions. Of note, women tend to rate their health as poor and very poor and also have a higher UI prevalence.

Evidence points towards a connection between COPD and increased UI prevalence [17, 18]. Coughing spells in COPD can elevate abdominal pressure, potentially causing stress incontinence. Additionally, certain COPD medications can impact continence [19]. Consistent with existing literature, our study found an increased UI risk among individuals with COPD, with a higher risk among men. This finding underscores COPD’s potential contribution to susceptibility to UI.

Hypertension’s effects on women, including heightened nocturnal voiding and stress urinary incontinence, have been explored, but its broader influence on UI remains less understood [17, 20]. In our study, we identified significant differences in UI prevalence between hypertensive and non-hypertensive individuals, highlighting a potential connection between blood pressure regulation and UI. This underscores the relevance of managing hypertension not just for cardiovascular well-being but also concerning UI prevention and management.

Diabetes emerges as an independent UI risk factor, unexplained by obesity [21, 22]. Our study substantiated this finding, identifying diabetes as a risk factor for UI in both genders, whereas obesity increased UI risk solely in women. The elevated UI prevalence among individuals with diabetes underscores the potential impact of blood sugar regulation on UI occurrence. Diabetic neuropathy, a common diabetes complication, can damage nerves that control bladder muscles [23]. Over time, high blood sugar levels can weaken bladder muscles, affecting storage capacity. Managing diabetes could potentially play a role in mitigating UI risk.

The relationship between obesity and UI has been well-studied [24–26]. Our findings align with prior research indicating that maintaining a healthy BMI could benefit UI prevention or treatment. BMI was identified as a significant risk factor for UI in women, with obese women exhibiting a twofold UI risk increase. The complex interplay between obesity and UI mechanisms is not entirely comprehended, but Swenson et al.‘s [26] study suggest that increased intravesical pressure in obese women could elevate UI risk due to heightened demands on continence mechanisms.

Our results also highlight the relevance of an active lifestyle. Sedentary behavior was associated with higher UI risk, while physical activity correlated with lower risk. Numerous studies emphasize the positive impact of physical activity on UI risk reduction among women of varying age groups [27–29]. Our findings, however, indicate no such association between physical activity, sedentary behavior, obesity, and UI in men. Nonetheless, other studies suggest that physical activity and obesity could influence postprostatectomy UI [30].

The present study provides valuable insights into the prevalence and associated factors of urinary incontinence in Turkiye. We utilized data from the 2019 Turkish Health Survey (THS), which included a nationally representative sample of 9,740 households. This extensive sample size enhances the generalizability of findings to the broader Turkish population.

However, this study has some limitations that should be considered when interpreting the results. The cross-sectional design restricts the establishment of causal relationships and long-term trends. Reliance on self-reported data introduces potential recall and social desirability biases, impacting the accuracy of variables such as UI prevalence, chronic conditions, and health-related behaviors. The study’s limited temporal scope, focusing on the past 12 months, may not capture broader trends. On the other hand, questioning the past 12 months prevent recall bias.

The use of secondary data also imposes certain limitations to the study’s scope, constrained by available variables and their predefined definitions. This limitation hinders the examination of additional factors and refinement of measurements. For instance, the Yes/No format of the urinary incontinence (UI) question may not fully capture the nuanced nature of UI experiences, lacking specificity on types like urge and stress incontinence. Translation concerns, particularly regarding the phrase “problems controlling bladder,” may lead to an overestimation of UI incidence.

Furthermore, a fundamental limitation is the lack of detailed information on various etiological factors related to urinary incontinence (UI) for both men and women in the available dataset. The absence of specific data on gender-specific etiologies, such as the impact of prostate interventions on male UI, is recognized as a constraint, impeding a comprehensive exploration of the diverse factors contributing to UI in both male and female populations.

## Conclusion

This comprehensive study on UI in both men and women has shed light on significant gender disparities and determinants of UI. Notably, UI affects approximately 9% of the overall population, with a marked difference between genders. Importantly, this gender difference decreases with age, highlighting the evolving nature of UI across the lifespan.

One striking finding is the role of modifiable and non-modifiable causes in the prevalence of UI. Marital status and employment status, for instance, emerge as significant determinants. Separated, divorced, or widowed individuals, particularly women, have higher rates of UI, indicating a possible connection between emotional and social factors and UI. Employment status also matters, with employed men having lower rates and retired women facing higher rates. These findings suggest addressing marital and employment-related stressors might help alleviate UI, especially in women.

Furthermore, modifiable factors such as obesity, physical inactivity, and smoking are associated with higher UI prevalence, primarily in women. This highlights the importance of lifestyle modifications and targeted interventions to reduce UI risk, especially in the female population. Conversely, non-modifiable factors like age and the presence of chronic conditions such as COPD, hypertension, and diabetes are more strongly associated with UI in men. This underscores the need for tailored healthcare approaches for older men and those with underlying medical conditions.

In summary, our study underscores that UI is not a one-size-fits-all condition. It is influenced by a complex interplay of modifiable and non-modifiable factors that vary between men and women. This nuanced understanding should encourage further research to develop more precise and effective strategies for preventing and managing UI in both genders, ultimately reducing the overall burden of this condition on public health.

## Data Availability

The research data utilized in this study was derived from the 2019 micro dataset of the Turkey Health Survey, which was administered by the TurkStat, the Turkish Statistical Institute. TurkStat conducts this survey at regular intervals using a face-to-face methodology, sampling households to represent the entire country. To ensure the privacy and confidentiality of individuals, TurkStat collaborated with universities to anonymize the data, and graciously provided access to it for research purposes. For more detailed information regarding the Turkey Health Survey 2019 and the microdata used in this study, please visit the following link: https://evam.tuik.gov.tr/dataset/detail/28.
